# Chronic Myelomonocytic Leukemia (CMML) as a Rare Cause of Spontaneous Atraumatic Splenic Rupture in a 65-Year-Old Male

**DOI:** 10.7759/cureus.80357

**Published:** 2025-03-10

**Authors:** Mohammad N Sabbagh, Blake Hoerdeman, Reza Hooshyar, Ijeoma Ejeh

**Affiliations:** 1 Student Research Connect, Edward Via College of Osteopathic Medicine (VCOM), Blacksburg, USA; 2 Surgery, Cape Fear Valley Medical Center, Fayetteville, USA

**Keywords:** acute care surgery and trauma, chronic myelomonocytic leukemia (cmml), spleen, spleen hemorrhage, spleen pathology, spleen rupture

## Abstract

This report describes a patient with a history of chronic thrombocytopenia who presented with splenic rupture and hemorrhagic shock in the absence of trauma. Four days earlier, the patient had visited the emergency department with symptoms of sore throat, fever, and myalgia. Due to the relatively benign presentation, further workup was deferred in favor of a presumed infectious etiology. This delay in diagnosis led to the patient presenting with life-threatening injuries, necessitating an emergent splenectomy. Subsequent evaluation revealed an underlying diagnosis of chronic myelomonocytic leukemia (CMML), which ultimately caused the splenic rupture. This case underscores the importance of considering hematologic malignancies, including rare pathologies like CMML, in the differential diagnosis of atraumatic splenic rupture (ASR).

## Introduction

Atraumatic splenic rupture (ASR) is a rare and life-threatening condition with limited data on risk factors, outcomes, and management strategies [1,2]. Most cases of ASR are caused by one of three conditions: malignant hematological diseases, such as acute leukemia or lymphoma; infectious diseases, like mononucleosis and malaria; and local inflammatory disorders, such as acute or chronic pancreatitis. Certain medications, particularly anticoagulants and antiplatelet agents, have also been implicated in ASR. A systematic review of over 600 publications classified ASR into two main categories: the majority (93%) were attributed to a pathologic etiology, as seen in our case report, while a smaller proportion (7%) were classified as idiopathic [3]. As research in this field advances, additional underlying pathologies contributing to ASR may be identified, similar to what was observed in our case. 

Here, we present the case of a 65-year-old male who arrived at the emergency department in hemorrhagic shock secondary to a ruptured spleen. After an emergent exploratory laparotomy and splenectomy, the patient was diagnosed with chronic myelomonocytic leukemia (CMML), which was identified as the underlying cause of the splenic rupture.

## Case presentation

A 65-year-old male with a medical history of hypertension, hyperlipidemia, and chronic obstructive pulmonary disease presented to an outside hospital ED with complaints of sore throat, fever, and myalgia. Initial blood evaluation revealed an elevated WBC count of 44 x 10³/µL and a decreased platelet count of 44 x 10³/µL. Further investigation of these abnormalities was deferred as the patient was otherwise stable and asymptomatic. The leukocytosis was attributed to a likely infectious etiology, while the thrombocytopenia was a chronic issue, known to the patient for many years. Tests for streptococcal pharyngitis, COVID-19, and infectious mononucleosis were negative. The patient was discharged with a short course of antibiotics and asked to follow up with his primary care physician.

**Figure 1:**
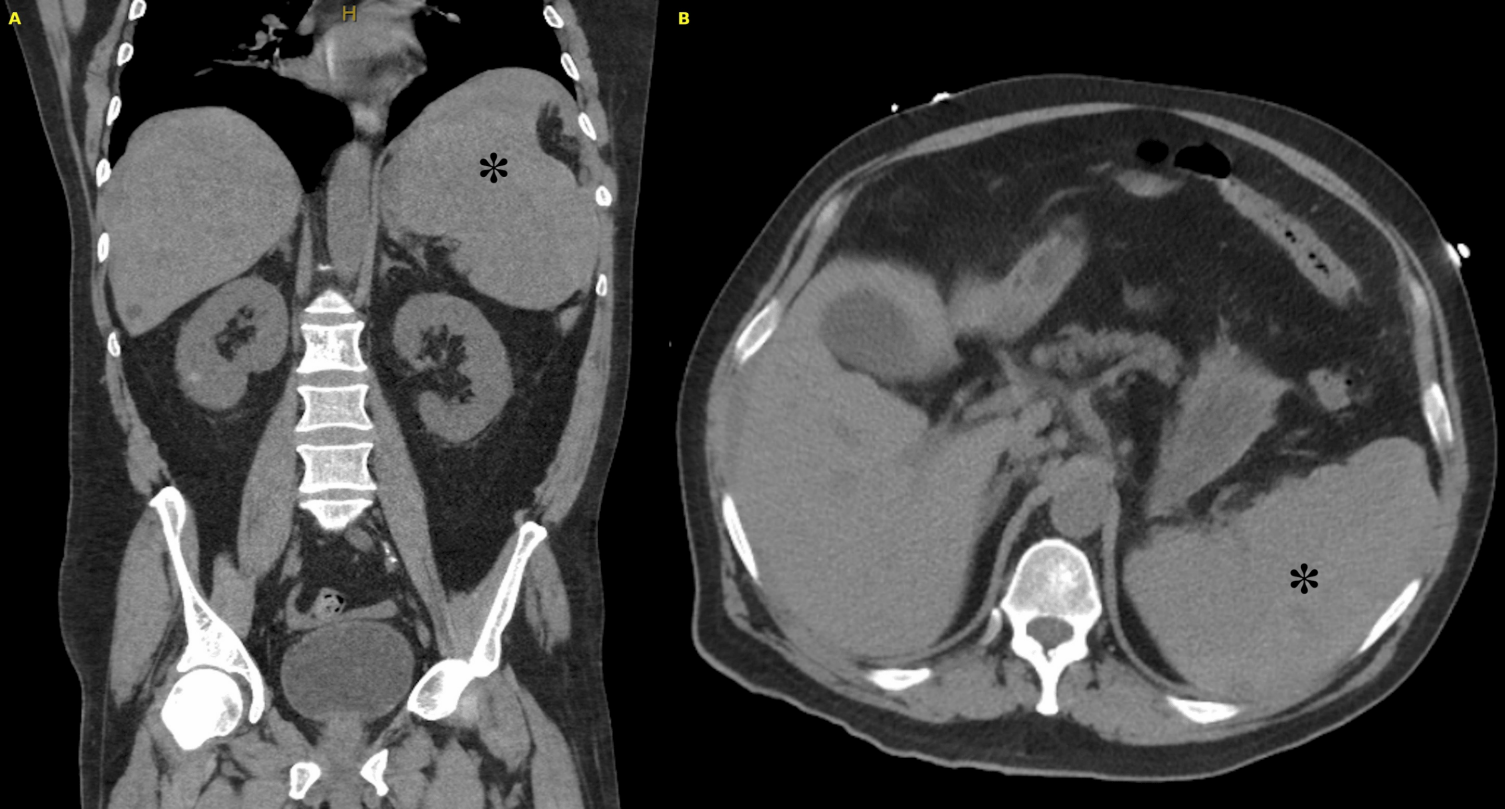
Preoperative CT images reveal markedly abnormal findings, with a large perisplenic hematoma obscuring differentiation between blood products and splenic parenchyma. Panel A (left) displays a coronal view of the spleen, while panel B (right) shows an axial view. An asterisk marks the ruptured spleen and the associated hematoma.

Four days later, the patient returned to the same ED with new complaints of generalized weakness and a syncopal episode. He was afebrile, with a heart rate of 92, and markedly hypotensive at 57/39 mmHg. The patient was found to be hypovolemic and nearing complete respiratory collapse. Resuscitation was initiated with 2 liters of crystalloid fluids and 2 units of packed RBCs (PRBC). A CT scan of the abdomen and pelvis revealed significant splenomegaly, splenic rupture, and hemoperitoneum (Figure 1). Blood results revealed a persistently increased WBC count (57 x 10³/µL), decreased hemoglobin (10 g/dL), decreased platelet count (63 x 10³/µL), and an elevated lactate (3 mmol/L). Given the splenic rupture and persistent hemodynamic instability, the patient was transferred to our institution via helicopter for a higher level of care.

Upon arrival at our ED, the patient was diaphoretic and hypotensive, with a blood pressure of 70/30 mmHg. An arterial line was placed, and resuscitation with 2 additional units of PRBC was initiated. A brief history revealed that the patient began experiencing progressively worsening abdominal pain in the left upper quadrant for several days prior to his syncopal episode and presentation. The patient continued to be hemodynamically unstable despite multiple resuscitation attempts and was emergently taken to the operating room for an exploratory laparotomy and splenectomy.

The abdomen was entered using a midline laparotomy incision, and dissection was carried down to the underlying fascia using electrocautery. Immediately upon entering the abdominal cavity, a large volume of hemoperitoneum was encountered, and 2400 mL of hemoperitoneum was evacuated. The massive transfusion protocol was initiated at this time. Dense adhesions around the spleen were carefully taken down. The spleen, found deep and posterior in the left upper quadrant, was surrounded by blood clots and partially fragmented. The spleen was carefully removed in multiple fragments and sent for pathologic evaluation.

Two intra-abdominal drains were placed, one in the pelvis and one adjacent to the splenectomy site. The abdomen was then closed in a typical fashion. By the conclusion of surgery, the patient had received a total of 12 units of PRBC, 8 units of fresh frozen plasma, 5 units of platelets, and 2 units of cryoprecipitate. The patient remained intubated and was transferred to the intensive care unit in stable condition.

Postoperatively, the patient was successfully weaned off vasopressor support and extubated by postoperative day (POD) 2. The patient’s postoperative recovery lasted 12 days and was uncomplicated, except for persistently abnormal WBC (ranging from 22-40) and fluctuating platelet counts (ranging from 18-176). After his extubation, a more detailed history revealed a 10-year history of easy bruising and thrombocytopenia. He had last followed up with a hematologist a year prior, at which time he was presumed to have chronic immune thrombocytopenia but was lost to follow-up. A chart review from his prior hematology visit revealed no abnormalities, except for low platelets at 67 x 10³/µL. Notably, his WBC had been normal at 7 x 10³/µL, and prior imaging showed no concern for splenomegaly. On POD 12, after receiving vaccinations for encapsulated organisms, the patient was discharged to a subacute rehab facility and scheduled to follow up with the surgical and oncological teams that had managed his care during his hospitalization.

The pathology of the spleen revealed extramedullary hematopoiesis and an abnormal monocytic infiltrate. This corresponds with the abnormal CD56-positive monocytes noted in the patient’s peripheral blood smear. Flow cytometry analysis revealed a small population of circulating myeloid blasts (0.10% of total WBC), an increased number of granulocytes with a marked left shift (48.4% of total WBC), and increased mature monocytes (47.1% of total WBC), with a significant subset aberrantly expressing CD56. B cells demonstrated no evidence of clonality or antigenic aberrancy, and the T cells were immunophenotypically normal. These findings, along with the pathologic sample of the spleen, are most compatible with CMML.

## Discussion

Splenic rupture can be categorized into either atraumatic or traumatic, with traumatic rupture being more common. Blunt abdominal trauma, frequently due to motor vehicle collisions, is the leading cause of traumatic splenic rupture. The treatment of traumatic splenic rupture is typically divided into operative and nonoperative management (NOM). In recent years, there has been an effort to preserve the hematologic and immunologic functions of the spleen, with NOM becoming the gold standard in management [4].

Splenic artery embolization can be used as an adjunct to NOM and has shown much success, although there remains some debate regarding its indications and best use [5]. The management of traumatic splenic injury with NOM is well-studied, and established guidelines allow clinicians to identify factors that predict NOM success or indicate the need for immediate splenectomy [6-9].

In contrast, ASR is not as well characterized, and there is no consensus regarding its optimal management. One reason ASR may be underreported and poorly characterized in the literature is the inconsistent and sometimes misleading terminology used to describe it [2,3]. For example, “atraumatic” and “spontaneous” are often used interchangeably, although “spontaneous” can refer to a splenic rupture after a minor or unnoticed trauma, which differs from a truly “atraumatic” rupture where underlying pathology, like extramedullary hematopoiesis, is the cause [3].

ASR carries an overall 12% mortality rate and can reach 21% in patients with neoplastic disorders, as is the case with our patient [3,10]. Mortality rates can vary based on factors such as age, comorbidities, injury severity, or, as in ASR cases, the underlying pathology. However, trauma-related splenic ruptures tend to have a lower mortality rate. For instance, a multi-institutional study on blunt abdominal trauma found that mortality rates varied by center. One center, which performed surgery on a higher proportion of patients (66.7%) with more severe splenic injuries and a reported average age of 40, reported a mortality rate of 15.3%. In contrast, a center with a younger patient population (average age of 26.1) and less severe injuries reported a much lower proportion of surgical management (6.9%) and a mortality rate of 3.6% [9].

The indications for splenectomy in ASR management are often extrapolated from those identified in cases of blunt splenic injury. However, there is a notable difference in practice, with 86.5% of ASR cases treated with splenectomy, either primarily or after failed NOM, compared to only 40-50% of traumatic splenic injury cases historically requiring splenectomy [9]. These findings suggest that delays in diagnosis of ASR may lead to more advanced disease upon presentation and necessitate more urgent and invasive management, such as splenectomy.

While hematologic malignancy is a recognized risk factor for ASR, acute leukemias are more commonly reported, with CMML rarely cited [3]. Thus, the case of ASR secondary to CMML seen in our patient can be considered unique. Furthermore, his presentation was atypical, initially resembling a common viral or bacterial infection. He lacked classic symptoms of splenic rupture, such as acute abdominal pain or hemodynamic instability, which, if present, may have prompted earlier imaging. Unlike cases of blunt abdominal trauma, where imaging is routinely performed, allowing for early identification and consideration of NOM, his initial presentation did not warrant immediate imaging. This diagnostic ambiguity likely contributed to a delayed diagnosis, ultimately necessitating more invasive management, such as surgery. This case underscores the need for greater awareness of atypical ASR presentations. Future studies should focus on refining and standardizing ASR terminology, with the goal of developing guidelines akin to those established for cases of traumatic splenic injury.

## Conclusions

ASR remains a poorly characterized condition, with inconsistent terminology complicating its diagnosis and management. While hematologic malignancies such as acute leukemias are known to cause ASR, CMML is rarely reported. This case highlights the complexity of diagnosing ASR, especially given our patient’s acute presentation and vague symptoms. Likewise, the increased frequency of splenectomy in ASR, compared to traumatic splenic injury, underscores the need for heightened clinical suspicion and specific management guidelines. Future efforts should first focus on the use of clear terminology with the goal of developing management guidelines specifically for cases of ASR.
